# Relationship Between Serum ω-3 Polyunsaturated Fatty Acid Concentration and Fatty Acid Fraction of Epicardial Adipose Tissue in Patients With Cardiovascular Disease

**DOI:** 10.7759/cureus.73417

**Published:** 2024-11-11

**Authors:** Taira Fukuda, Toshiaki Nakajima, Takaaki Hasegawa, Hirohisa Amano, Takuo Arikawa, Ikuko Shibasaki, Mitsuo Ohni, Shichiro Abe, Masashi Sakuma, Hirotsugu Fukuda, Shigeru Toyoda

**Affiliations:** 1 Department of Liberal Arts and Sciences, Kanagawa University of Human Services, Yokosuka, JPN; 2 Department of Cardiovascular Medicine, Dokkyo Medical University, Mibu, JPN; 3 Department of Medical KAATSU Training, Dokkyo Medical University, Mibu, JPN; 4 Department of Cardiovascular Surgery, Dokkyo Medical University, Mibu, JPN; 5 Department of Geriatric Medicine, Kyorin University School of Medicine, Mitaka, JPN

**Keywords:** cardiovascular surgery, docosahexaenoic acid, eicosapentaenoic acid, epicardial adipose tissue, vascular inflammation

## Abstract

Background: ω-3 polyunsaturated fatty acids (PUFAs), such as eicosapentaenoic acid (EPA, C20:5ω3) and docosahexaenoic acid (DHA, C22:6ω3), are widely regarded as cardioprotective. EPA, but not DHA, has been reported to prevent fibrosis in heart failure. The relationship between the ω-3 PUFA fraction in epicardial adipose tissue (EAT) and subcutaneous adipose tissue (SAT) and vascular inflammation in patients with cardiovascular disease remains unclear.

Methods: EAT was collected from patients undergoing cardiovascular surgery (n=21, 11 men, 10 women, 70.4±9.0 years old). Fatty acid fractions were measured in serum, SAT, and EAT by gas chromatography, and serum tumor necrosis factor (TNF) α was measured. No patient had taken EPA or DHA supplements.

Results: DHA concentrations were significantly higher in EAT than in SAT (p=0.001); EPA showed no significant difference. The EPA level of EAT correlated with the serum EPA concentration (p=0.009) but did not correlate significantly with serum DHA, α-linolenic acid (ALA, C18:3ω3), or linoleic acid (LA, C18:2ω6) concentrations. The EPA level of EAT showed a strong correlation with docosapentaenoic acid (DPA, C22:5ω3) and the DHA level of EAT (DHA: p<0.001). ALA (r=-0.519, p=0.039) and EPA (r=-0.611, p=0.027) levels of EAT correlated negatively with the serum TNFα concentration. There were no significant differences in EPA in serum, SAT, and EAT between patients with and without atherosclerotic heart disease.

Conclusions: Among ω-3 PUFA fractions in EAT, ALA and EPA were associated with anti-inflammatory effects in patients with cardiovascular disease. It is likely that an increase in serum EPA concentration is needed to increase ω-3 PUFA levels in EAT.

## Introduction

Numerous reports have shown that unsaturated fatty acids, such as fish oil, suppress cardiovascular events [1]. ω-3 polyunsaturated fatty acids (PUFAs), eicosapentaenoic acid (EPA, C20:5ω3) and docosahexaenoic acid (DHA, C22:6ω3), are essential fatty acids found in fish oil. The importance of ω-3 PUFAs in coronary artery disease (CAD) is well known, and serum EPA levels have been reported as predictive factors in the secondary prevention of acute myocardial infarction [2] and heart failure [3]. In a clinical trial in patients with New York Heart Association (NYHA) class II-IV heart failure, the ω-3 PUFA group had significantly reduced all-cause mortality and cardiovascular hospitalizations compared to the placebo group [4]. Both European and American heart failure guidelines recommend the intake of ω-3 PUFAs as adjunctive therapy, although recommendations vary [5,6].

Among the ω-3 PUFAs, EPA, but not DHA, has been reported to prevent fibrosis in heart failure [7]. Heart diastolic dysfunction often follows heart interstitial fibrosis and is common in heart failure with preserved ejection fraction (HFpEF). In one animal model of heart diastolic dysfunction, EPA- and DHA-specific diets were administered to examine myocardial remodeling following pressure overload. Results showed that EPA, but not DHA, inhibited fibrosis following pressure overload [7]. Epicardial adipose tissue (EAT) releases adipocytokines, such as tumor necrosis factor alpha (TNFα), which play an important role in cardiovascular disease [8]. In our previous study, EAT samples were collected from patients undergoing cardiovascular surgery, and serum TNFα levels were inversely correlated with epicardial α-linolenic acid (ALA, C18:3ω3) content, suggesting that ω-3 PUFA content, such as ALA in EAT, is associated with anti-inflammation [9]. However, the relationship between ω-3 PUFA fractions, such as EPA and DHA, and vascular inflammation in EAT and subcutaneous adipose tissue (SAT) in patients with cardiovascular disease remains unclear.

In the present study, we aimed to determine the relationships among ω-3 PUFA fractions in serum, EAT, and SAT in patients with cardiovascular disease and the relationship between ω-3 PUFAs in serum, EAT, and SAT and vascular inflammation.

## Materials and methods

Participants

From November 2015 to June 2016, we evaluated 21 patients who underwent cardiovascular surgery at Dokkyo Medical University Hospital. The protocol was approved by the local ethics committee of Dokkyo Medical University Hospital (No. 27074). No patient took EPA or DHA supplements.

Fasting venous blood samples were obtained in polystyrene tubes with and without sodium ethylenediaminetetraacetic acid (1 mg/mL) and without anticoagulant. Serum and plasma were immediately separated by centrifugation at 3000 rpm, 4°C, for 10 minutes. Fasting blood glucose (FBG), total cholesterol, hemoglobin A1c, brain natriuretic peptide, low-density lipoprotein cholesterol, high-density lipoprotein cholesterol, and estimated glomerular filtration rate (eGFR) were measured. The eGFR was calculated with the following equation:

eGFR (mL/min/1.73m^2^) = 194 × serum creatinine-1.094 × age-0.287 (men)

eGFR (mL/min/1.73m^2^) =194 × serum creatinine-1.094 × age-0.287 × 0.739 (women)

FBG and biochemical data were analyzed by routine biochemical methods in the clinical laboratory of Dokkyo Medical University Hospital. Levels of high-sensitivity C-reactive protein (hs-CRP), a marker of inflammation, were measured by latex-enhanced nephelometric immunoassay (N Latex CRP II and N Latex SAA, Dade Behring Ltd., Tokyo, Japan).

Luminex assay

To measure fasting serum TNFα levels, peripheral venous blood was drawn into EDTA-free and pyrogen-free tubes on the morning of cardiovascular surgery. Serum was stored at -80°C in small-volume storage tubes. The Luminex assay was applied to determine serum levels of TNFα. Serum concentrations of TNFα were calculated by comparing assay readings on a Luminex 200TM system (Luminex Co., Austin, TX, USA). The detection threshold was 1.2 pg/mL.

Adipose tissue collection

Adipose tissue samples were obtained as previously described [9,10] prior to the start of cardiovascular surgery. The EAT samples (average 0.5-1.0 g) were collected near the proximal right coronary artery. The SAT samples were obtained from around the xiphoid process of the sternum. Samples were stored at -80°C for later analysis.

Determination of the fatty acid composition

Serum Fatty Acids

Approximately 0.2 mL of serum samples and 2 mL of chloroform-methanol (2:1) solution (1 mL of water, 666 μL of methanol, 333 μL of chloroform) were placed in a Pyrex centrifuge tube and homogenized with Polytron (PCU2-110, KINEMATICA GmbH, Switzerland), homogenized, and then centrifuged at 3000 rpm for 10 min. Portions of the chloroform-methanol extracts were transferred to separate Pyrex tubes and dried under nitrogen gas vapor. Dried samples were dissolved in 100 µL of 0.4 M potassium methoxide-methanol/14% boron trifluoride-methanol solution. The fatty acid concentration of the solution was determined by gas chromatography (Shimazu GC 17A, Kyoto, Japan) at SRL Corporation. Fatty acid content was expressed as a percentage of total fat extracted for each fatty acid. These values were then converted to mass by multiplying the percentage of fat by the mass of fat per gram of tissue (μg/g).

Fatty Acid Composition of Adipose Tissue

Fat was extracted from approximately 200-250 mg of adipose tissue. Fat tissue and 100 μL of water and 1 mL of chloroform-methanol (2:1) solution (666 μL of methanol and 333 μL of chloroform) were placed in polypropylene centrifuge tubes. The samples were homogenized in a disperser (TT-10 basic ULTRA-TURRAX®, IKA®, Wilmington, DE, USA) and then centrifuged at 2000 rpm for 5 min. The entire supernatant of the chloroform-methanol extract in the upper layer was transferred to another polypropylene tube. The remaining material and 1.52 mL of chloroform-methanol solution (800 μL of methanol, 400 μL of chloroform, 320 μL of water) were again placed in a polypropylene centrifuge tube, homogenized in a disperser, and then centrifuged at 2000 rpm for five minutes. The entire supernatant of the chloroform-methanol extract was added to the polypropylene tube containing the first extract and mixed with 1.4 mL of chloroform solution (0.7 mL of water, 0.7 mL of chloroform) and then centrifuged at 2000 rpm for five minutes. The lower layer containing fat was transferred to a glass container and stored at 37°C overnight to allow the chloroform to evaporate. The fat was applied to a gas-liquid chromatograph (SRL Corporation) as described above.

Statistical analysis

All data are presented as mean ± standard deviation or percentage (categorical data). After testing normality, associations between parameters were analyzed by the Pearson method for normally distributed parameters and the Spearman method for non-normally distributed parameters. Comparisons between groups were performed using independent data t-tests or Mann-Whitney U tests. Among patients with valvular disease, those with aortic valve stenosis were included in the atherosclerotic group [11]. Comparisons within groups were made by Wilcoxon's signed rank difference test. IBM SPSS Statistics for Windows, Version 28 (Released 2021; IBM Corp., Armonk, New York, United States) was used for statistical analysis. A p-value < 0.05 was considered statistically significant.

## Results

**Figure 1:**
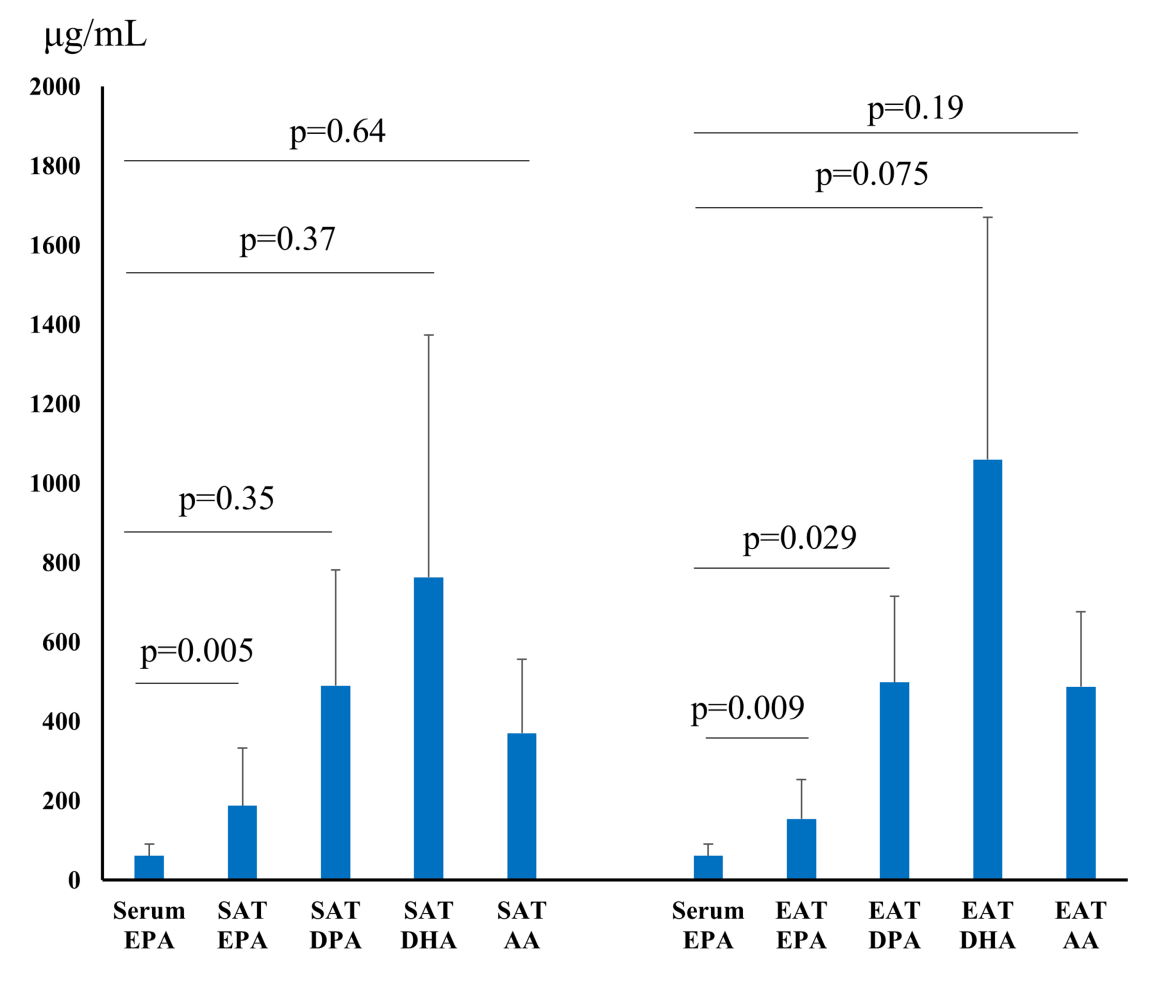
Correlation between serum EPA concentration and fatty acid content of subcutaneous adipose tissue and epicardial adipose tissue. SAT, subcutaneous adipose tissue; EAT, epicardial adipose tissue; EPA, eicosapentaenoic acid; DPA, docosapentaenoic acid; DHA, docosahexaenoic acid; AA, arachidonic acid.

Baseline patient characteristics are summarized in Table 1. The mean age was 70.4 ± 9.0 years, and body mass index (BMI) was 26.0 ± 5.1 kg/m^2^. The serum EPA concentration showed a strong correlation with the EPA level of SAT (Figure 1, p=0.005), but not with the docosapentaenoic acid (DPA, C22:5ω3) level. The serum EPA concentration did not correlate with the DHA or arachidonic acid (AA, C20:4ω6) level of SAT. The serum EPA concentration was strongly correlated with the EPA level of EAT (p=0.009) and with the DPA level (p=0.029). The serum EPA concentration did not correlate with the DHA or AA level of EAT. The EPA level of EAT did not correlate significantly with serum DHA, ALA, or linoleic acid (LA, C18:2ω6) concentrations.

**Table 1:** Patient characteristics. Mean ± SD values or percentage (categorical data) are shown. BMI, body mass index; NYHA, New York Heart Association; HbA1c, hemoglobin A1c; eGFR, estimated glomerular filtration rate; HDL, high-density lipoprotein; LDL, low-density lipoprotein; hsCRP, high-sensitivity C-reactive protein; BNP, brain natriuretic peptide; TNFα, tumor necrosis factor α; LA, linoleic acid; ALA, α-linolenic acid; AA, arachidonic acid; EPA, eicosapentaenoic acid; DPA, docosapentaenoic acid; DHA, docosahexaenoic acid; CABG, coronary artery bypass grafting.

Number of patients	21		
Male, Female, %	52.4, 47.6	BNP, pg/mL	466 ± 882
Age, y	70.4 ± 9.0	TNFα, pg/mL	3.6 ± 2.7
BMI, kg/m^2^	26.0 ± 5.1	Serum LA, μg/mL	570.5 ± 155.1
Risk factors, %		Serum ALA, μg/mL	18.1 ± 7.9
Hypertension	81.0	Serum AA, μg/mL	155.3 ± 44.2
Diabetes	33.3	Serum EPA, μg/mL	61.1 ± 29.8
Dyslipidemia	52.4	Serum DPA, μg/mL	15.4 ± 6.2
Smoking	4.8	Serum DHA, μg/mL	114.1 ± 32.1
Hemodialysis	14.3	EPA/AA	0.42 ± 0.23
NYHA class	2.3 ± 1.1	Coronary artery disease, %	
Laboratory blood data		0-vessel disease	47.6
HbA1c, %	6.0 ± 0.7	1-vessel disease	0
Fasting blood glucose, mg/dL	112 ± 31	2-vessel disease	9.5
eGFR, mL/min/1.73m^2^	56 ± 28	3-vessel disease	42.9
Total cholesterol, mg/dL	173 ± 42	Cardiovascular surgery, %	
Triglycerides, mg/dL	125 ± 73	CABG	52.4
HDL cholesterol, mg/dL	51 ± 15	Valve replacement/repair	47.6
LDL cholesterol, mg/dL	98 ± 32	Others	28.6
hsCRP, mg/dL	0.28 ± 0.40		

**Figure 2:**
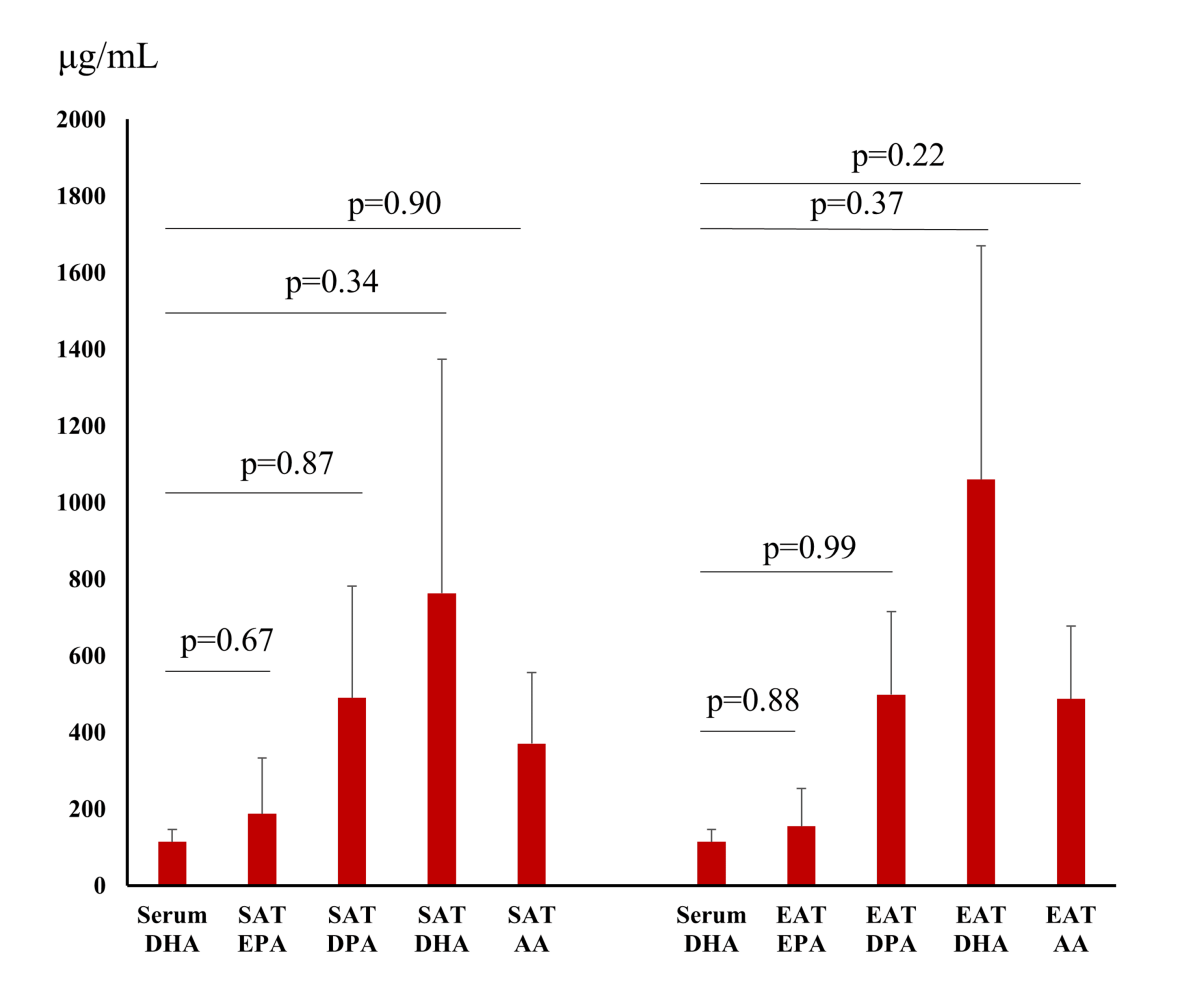
Correlation between serum DHA concentration and fatty acid content of subcutaneous adipose tissue and epicardial adipose tissue. SAT, subcutaneous adipose tissue; EAT, epicardial adipose tissue; EPA, eicosapentaenoic acid; DPA, docosapentaenoic acid; DHA, docosahexaenoic acid; AA, arachidonic acid.

The serum DHA concentration did not correlate with the EPA level of SAT, nor with the DPA level (Figure 2). The serum DHA concentration did not correlate with the DHA or AA level of SAT. The serum DHA concentration did not correlate with the EPA level of EAT, nor with the DPA level. The serum EPA concentration did not correlate with the DHA or AA level of EAT.

**Figure 3:**
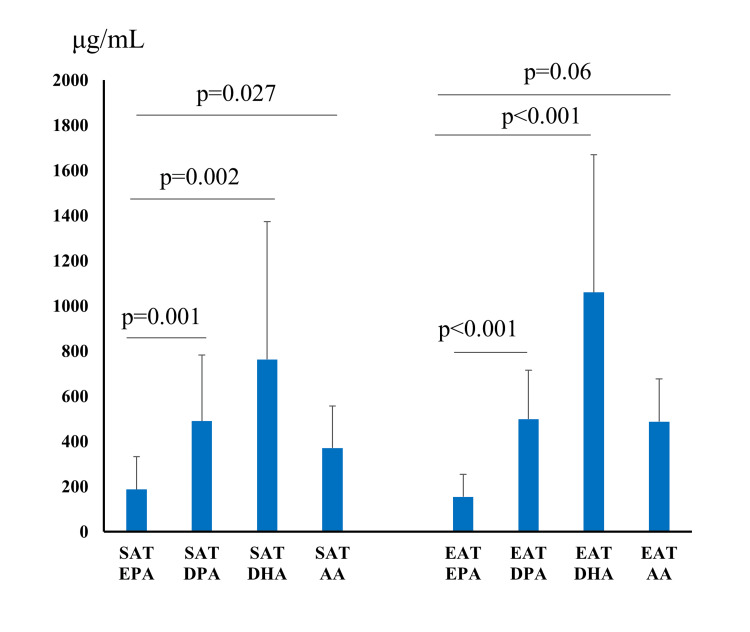
Correlation between EPA content and fatty acid content of subcutaneous adipose tissue and epicardial adipose tissue. SAT, subcutaneous adipose tissue; EAT, epicardial adipose tissue; EPA, eicosapentaenoic acid; DPA, docosapentaenoic acid; DHA, docosahexaenoic acid; AA, arachidonic acid.

The EPA level of SAT correlated strongly with the DPA and DHA levels of SAT (Figure 3, DPA: p=0.001; DHA: p=0.002) and with the AA level (p=0.027). The EPA level of EAT correlated strongly with the DPA and DHA levels of EAT (DPA: p<0.001; DHA: p<0.001), but not with the AA level.

**Figure 4:**
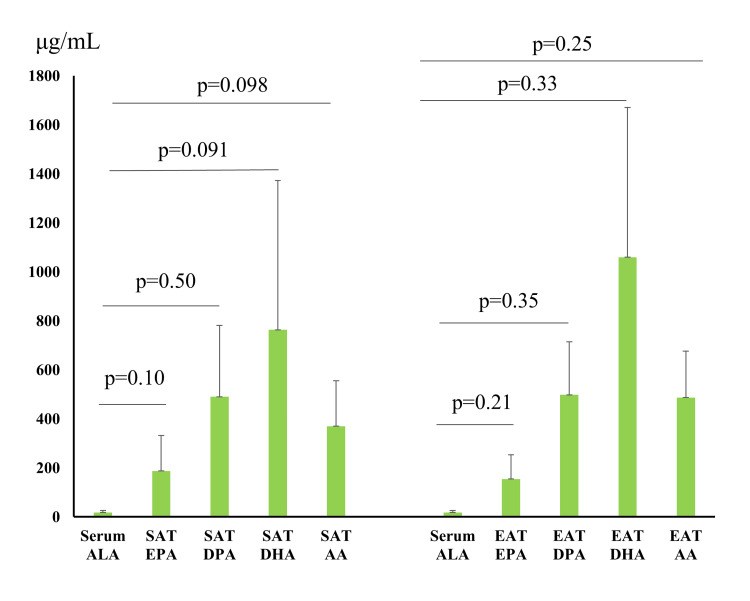
Correlation between serum ALA concentration and fatty acid content of subcutaneous adipose tissue and epicardial adipose tissue. SAT, subcutaneous adipose tissue; EAT, epicardial adipose tissue; ALA, α-linolenic acid; EPA, eicosapentaenoic acid; DPA, docosapentaenoic acid; DHA, docosahexaenoic acid; AA, arachidonic acid.

The serum ALA concentration did not correlate with the EPA level of SAT, nor with the DPA level (Figure 4). The serum ALA concentration did not correlate with the DHA or AA level of SAT. The serum ALA concentration did not correlate with the EPA level of EAT, nor with the DPA level. The serum ALA concentration did not correlate with the DHA or AA level of EAT.

**Figure 5:**
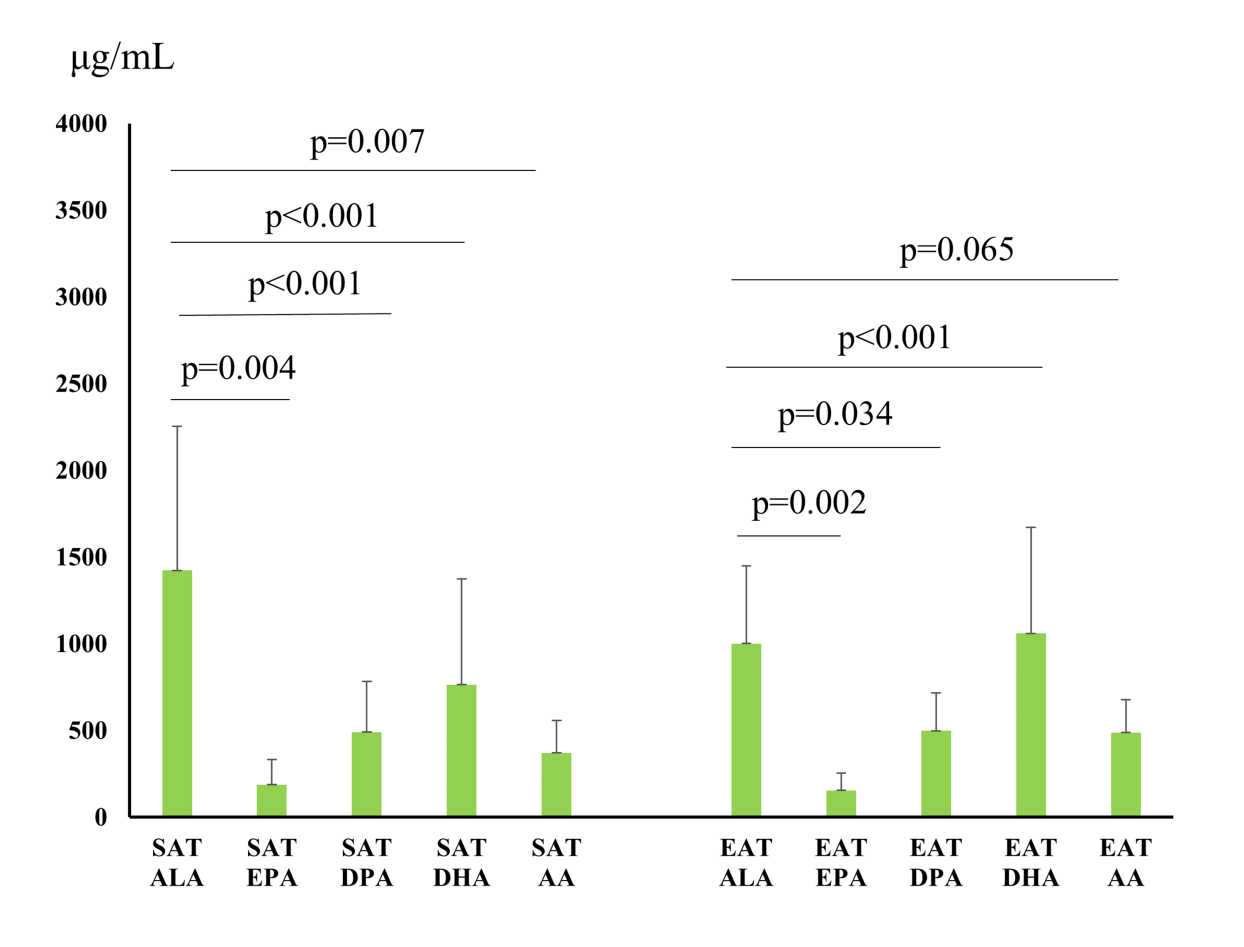
Correlation between ALA content and fatty acid content of subcutaneous adipose tissue and epicardial adipose tissue. SAT, subcutaneous adipose tissue; EAT, epicardial adipose tissue; ALA, α-linolenic acid; EPA, eicosapentaenoic acid; DPA, docosapentaenoic acid; DHA, docosahexaenoic acid; AA, arachidonic acid.

The ALA level of SAT correlated with the EPA, DPA, DHA, and AA levels of SAT (Figure 5, EPA: p=0.004; DPA: p<0.001; DHA: p<0.001; AA: p=0.007). The ALA level of EAT correlated with the EPA, DPA, and DHA levels of EAT (EPA: p=0.002; DPA: p=0.034; DHA: p<0.001), but not with the AA level.

**Figure 6:**
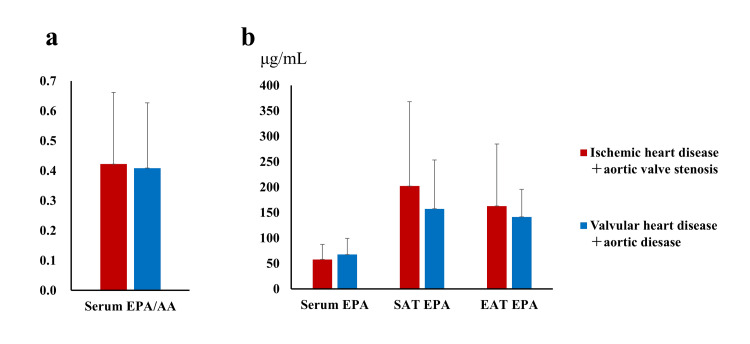
Comparison of (a) serum EPA/AA ratio, and (b) serum EPA concentration, EPA level of SAT, and EPA level of EAT between atherosclerotic and non-atherosclerotic disease groups. SAT, subcutaneous adipose tissue; EAT, epicardial adipose tissue; EPA, eicosapentaenoic acid; AA, arachidonic acid.

No significant differences in serum EPA concentration, serum EPA/AA ratio, EPA level of SAT, or EPA level of EAT were observed between the atherosclerotic (ischemic heart disease + aortic valve stenosis) and non-atherosclerotic (valvular heart disease + aortic disease) disease groups (Figure 6).

**Figure 7:**
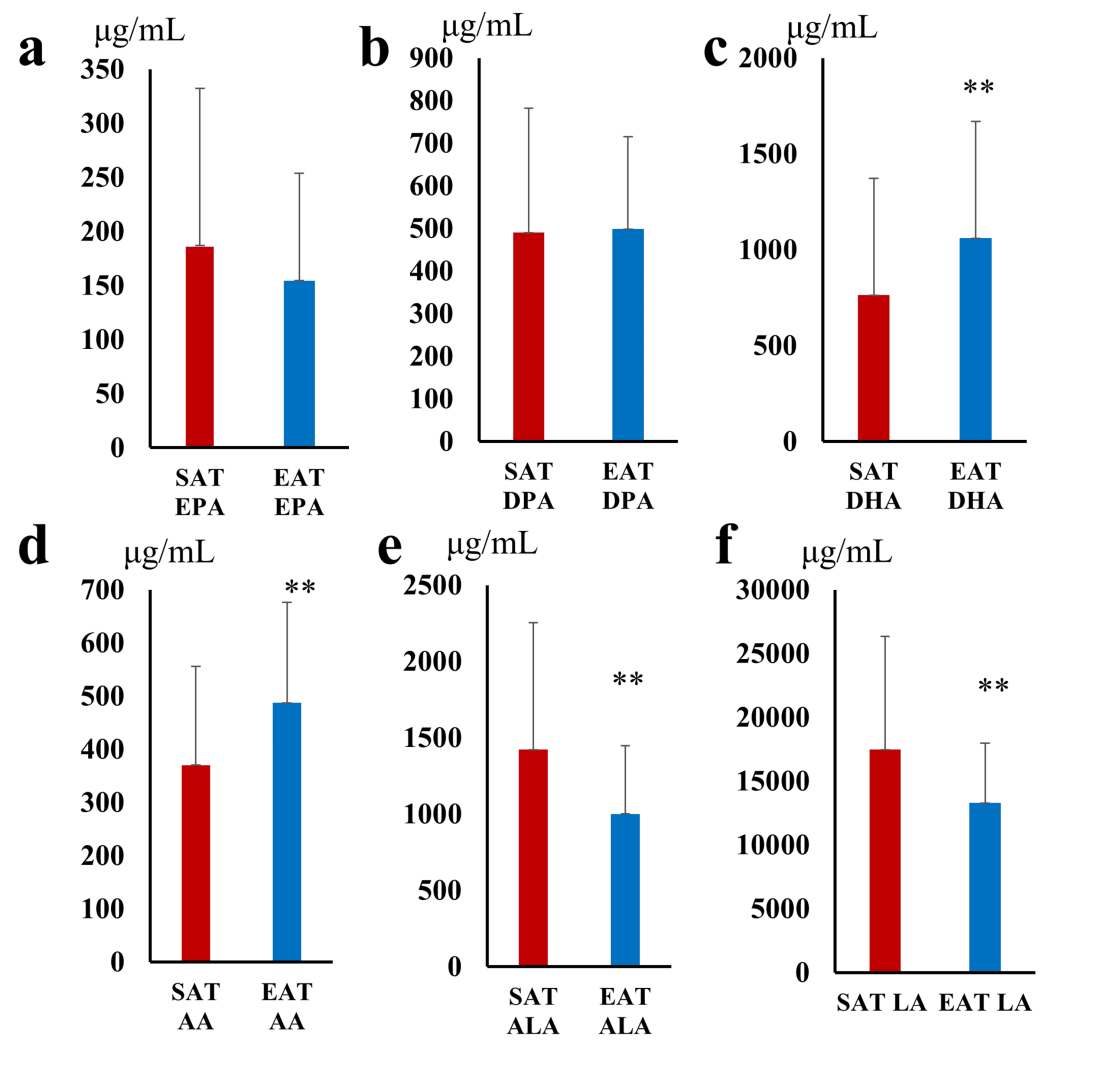
Comparison of (a) EPA, (b) DPA, (c) DHA, (d) AA, (e) ALA, and (f) LA between SAT and EAT. SAT, subcutaneous adipose tissue; EAT, epicardial adipose tissue; EPA, eicosapentaenoic acid; DPA, docosapentaenoic acid; DHA, docosahexaenoic acid; AA, arachidonic acid; ALA, α-linolenic acid; LA, linoleic acid. ** p<0.01.

No significant differences were found in EPA and DPA concentrations between SAT and EAT (Figure 7). DHA and AA concentrations were significantly higher in EAT (DHA: p=0.001; AA: p=0.008), while ALA and LA concentrations were significantly lower in EAT (ALA: p=0.002; LA: p=0.008).

**Figure 8:**
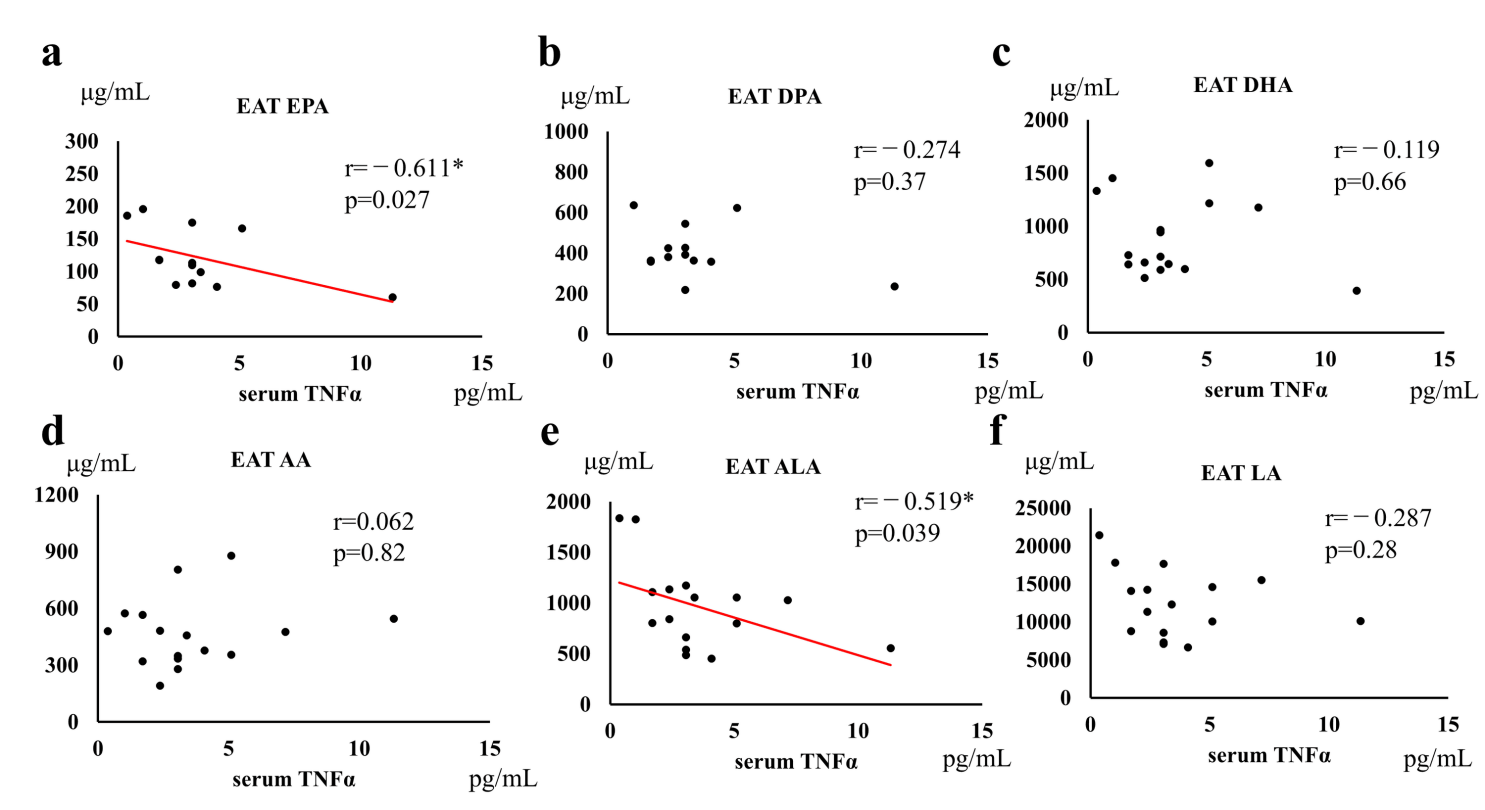
Correlation between (a) EPA, (b) DPA, (c) DHA, (d) AA, (e) ALA, and (f) LA content of EAT and serum TNFα levels. EAT, epicardial adipose tissue; TNFα, tumor necrosis factor α; EPA, eicosapentaenoic acid; DPA, docosapentaenoic acid; DHA, docosahexaenoic acid; AA, arachidonic acid; ALA, α-linolenic acid; LA, linoleic acid. * p<0.05.

The serum TNFα concentration was negatively correlated with the ALA and EPA levels in EAT (Figure 8; ALA: r=-0.519, p=0.039; EPA: r=-0.611, p=0.027), but not with the other fatty acid levels in EAT.

## Discussion

In the present study, the DHA level was significantly higher in EAT compared to SAT, but the EPA level showed no significant difference. The EPA level in EAT correlated with the serum EPA concentration, but not with the serum DHA, ALA, or LA concentration. The EPA level of EAT correlated strongly with the DPA and DHA levels of EAT. The ALA and EPA levels of EAT negatively correlated with the serum TNFα concentration.

Both LA and ALA, essential fatty acids, not only provide energy but also act as precursors of long-chain metabolites [12]. They have important physiological functions as substrates for the formation of eicosanoids, particularly prostaglandins, thromboxanes, and leukotrienes. The metabolic pathways of both causative fatty acids follow alternating stages of desaturation and chain elongation using the same set of enzymes. Thus, there is competition between the two fatty acid systems for their common enzymes. Even though Δ6-desaturase has a preference for ALA over LA, ALA is generally at a competitive disadvantage due to the higher dietary intake of LA. LA is metabolized to AA, a 20-carbon PUFA that is a precursor to eicosanoids, but the majority of recent experimental and clinical studies have evaluated the potential benefits of increasing dietary intake of LA [13]. Increasing dietary LA intake does not lead to a significant increase in AA tissue values or contribute to the biosynthesis of prostaglandins or other AA metabolites. This is because fatty acids can be metabolized by the elongase and desaturase enzymes to produce PUFAs with longer carbon chains and higher numbers of double bonds, but the conversion of LA to AA is reported to be very limited, less than 1% [13].

The first report on the association between fish oil and prevention of lifestyle-related diseases showed that the Inuit people of Greenland, a Danish territory, were less prone to myocardial infarction and other lifestyle-related diseases than mainland Danish people [14]. ω-3 PUFAs are effective in reducing cardiovascular events. ω-3 PUFA supplementation has been evaluated as an adjunctive therapy for cardiovascular disease and heart failure [15]. In patients with NYHA class II-IV heart failure, ω-3 PUFA supplementation is a reasonable adjunctive therapy to reduce mortality and cardiovascular hospitalizations [4,16]. Serum EPA/AA ratio is a useful biomarker in patients with CAD [17-19]. Iwamatsu et al. showed that the serum EPA/AA ratio was significantly lower in patients with acute coronary syndrome (ACS) than in those with chronic CAD or chest pain syndrome, while the serum DHA/AA ratio was comparable in both groups [17]. Multivariate logistic regression analysis using various coronary risk-related biomarkers discriminated ACS from other diseases, and the EPA/AA ratio was a significant independent predictor compared to the DHA/AA ratio [17]. These findings suggested that the EPA/AA ratio may be more closely related to the pathology of CAD, especially ACS and EPA intake may confer a greater advantage for plaque stabilization to prevent the development of ACS in CAD patients compared to DHA. Also, Abe et al. found that EPA treatment improves long-term prognosis in CAD patients with an EPA/AA ratio of 0.4 or less, and an EPA/AA ratio greater than 1.2 may be an appropriate EPA treatment target value to reduce mortality [18]. A recent prospective and multicenter study has shown that EPA treatment led to a numerically lower risk of coronary vascular events, but did not reach statistical significance in patients with stable CAD, a low EPA/AA ratio (<0.4), and on statin therapy [19].

EAT is an etiologic fat deposition associated with coronary atherosclerosis and cardiovascular events [8,20]. Sato et al. investigated the effect of EPA preparations on EAT. Using a multi-slice computed tomography scanner, they compared EAT and abdominal visceral adipose tissue (AVAT) volumes in 30 patients with CAD treated for six months [21]. When the control group (n=15, conventional treatment) and an EPA group (n=15, conventional treatment + 1800 mg purified EPA/day) were compared, results showed that AVAT and EAT volumes decreased in the EPA group but remained unchanged in the control group. Furthermore, changes in EAT and AVAT volumes were inversely correlated with changes in serum EPA levels, and an inverse correlation between EAT volume and serum EPA levels was observed, suggesting that intake of purified EPA was associated with a decrease in EAT and AVAT volumes.

The multifaceted effects of EPA, including its anti-inflammatory [22], anti-arrhythmic [23], platelet aggregating [24], and plaque stabilizing [25,26] actions, have been shown to reduce the incidence of cardiovascular events. It has been reported that the dietary ω-3 PUFA bind to free fatty acid receptor 4 (FFAR4, formerly GPR120), a protein located on the surface of adipocytes and muscle cells [27]. When ω-3 PUFA bind to this G protein-coupled receptor, FFAR4, it triggers a series of responses that ultimately protect the body from weight gain and inflammation. Eclov et al. found that EPA administration to cardiac fibroblasts did not cause EPA to accumulate in cardiac fibroblasts, but FFAR4 was found to be sufficient, and FFAR4 was required to inhibit transforming growth factor (TGF) β1-mediated fibrosis signaling, suggesting that EPA inhibits TGFβ1 signaling and fibrosis via FFAR4 in cardiac fibroblasts [7].

In patients with cardiovascular disease, dietary PUFA levels were inversely correlated with inflammatory markers, including C-reactive protein (CRP) and interleukin 1β (IL1β) [28]. An increase of 1 g of ω-6 or ω-3 PUFA in 1000 Kcal of diet resulted in an 8% or 48% decrease in IL1β, respectively. The conversion efficiency of plant-derived ALA to its longer-chain metabolite, EPA, ranges from 0.05-8% [12,29]. One study in a mouse model evaluated the cellular and molecular mechanisms of dietary (flaxseed oil) ALA in atherosclerosis [30]. The results showed that a high ALA diet reduced plaque area by 50% and decreased the T-cell content of plaques as well as the expression of vascular cell adhesion molecule-1 and TNFα. Both dietary ALA and direct ALA exposure limited T cell proliferation, differentiation, and inflammatory activity. Dietary ALA converted prostaglandin and isoprostane formation to ω-3 metabolites, potentially contributing to the atheroprotective effects of ALA. It is thus suggested that dietary ALA reduced experimental atherosclerosis, limited T cell-driven inflammation, and provided evidence for the theory that plant-derived ALA may provide a valuable substitute for marine ω-3 PUFAs. In the present study, not only EPA but also ALA in EAT was associated with anti-inflammatory effects in patients with cardiovascular disease. On the other hand, an increase in serum EPA concentration, but not serum ALA, was required to increase ω-3 PUFA levels in the EAT.

The present study provides new evidence relating the relationships between the ω-3 PUFA fraction in serum, EAT and SAT and vascular inflammation in patients with cardiovascular disease. However, there are some limitations as follows. First, it is likely that an increase of serum EPA concentration is needed to increase ω-3 PUFAs in EAT, but to clarify this possibility, it is necessary to examine whether the fatty acid fraction in EAT or SAT and the level of inflammatory cytokines changes when EPA is administrated. Second, in the present study, there were no significant differences in serum EPA concentration, serum EPA/AA ratio, EPA level of SAT, or EPA level of EAT between the atherosclerotic (ischemic heart disease + aortic valve stenosis) and non-atherosclerotic (valvular heart disease + aortic disease) disease groups. However, Shibasaki et al. reported that the mRNA levels of IL-6, IL-1β, monocyte chemoattractant protein-1, adrenomedullin, and leptin in the EPA were higher in patients with CAD than in those without CAD [10]. Thus, further studies using a larger number of patients are needed.

## Conclusions

Among the ω-3 PUFA in EAT, ALA and EPA were associated with anti-inflammatory effects in patients with cardiovascular disease. In addition, it is likely that increased serum EPA concentrations, but not serum DHA, ALA, and LA, were required to increase ω-3 PUFA levels in the EAT.
